# Gallic Acid and Diabetes Mellitus: Its Association with Oxidative Stress

**DOI:** 10.3390/molecules26237115

**Published:** 2021-11-24

**Authors:** Yu Xu, Guoyi Tang, Cheng Zhang, Ning Wang, Yibin Feng

**Affiliations:** School of Chinese Medicine, Li Ka Shing Faculty of Medicine, The University of Hong Kong, Hong Kong, China; xyzjh@hku.hk (Y.X.); tanggy@connect.hku.hk (G.T.); zttc@connect.hku.hk (C.Z.); ckwang@hku.hk (N.W.)

**Keywords:** diabetes mellitus, oxidative stress, inflammation, gallic acid

## Abstract

Diabetes mellitus (DM) is a severe chronic metabolic disease with increased mortality and morbidity. The pathological progression of DM is intimately connected with the formation and activation of oxidative stress (OS). Especially, the involvement of OS with hyperglycemia, insulin resistance, and inflammation has shown a vital role in the pathophysiological development of DM and related complications. Interestingly, accumulating studies have focused on the exploration of natural antioxidants for their improvement on DM. Of specific interest is gallic acid (GA), which is rich in many edible and herbal plants and has progressively demonstrated robust antioxidative and anti-inflammatory effects on metabolic disorders. To provide a better understanding of its potential therapeutic impacts and enhancement of human health care, the available research evidence supporting the effective antidiabetic properties of GA and relevant derivatives are needed to be summarized and discussed, with emphasis on its regulation on OS and inflammation against DM. This review aims to highlight the latest viewpoints and current research information on the role of OS in diabetes and to provide scientific support for GA as a potential antihypoglycemic agent for DM and its complications.

## 1. Introduction

Homeostatic disruption in host metabolism and the resultant hyperglycemia or insulin resistance act as the leading cause for diabetes mellitus (DM) development. Diabetic patients, segregated into two major types of type 1 and type 2 DM, are always associated with the long-term metabolic syndrome that is manifested at structural, physiological, and psychological levels of host organs, apparent with organs injury diseases including diabetic nephropathy, retinopathy, neuropathy, cardiomyopathy, and angiopathy. Diabetes and its associated complications are of significant scientific and clinical interest, due to their contribution to the rapid rise of noncommunicable diseases. Chronically sustained progressions of these micro-and macrovascular complications can be influenced by multiple pathogenic factors, above which oxidative stress (OS) is proved as a major trigger. Natural antioxidants with the protective effect against OS have attracted increasing interest in the development of antioxidant therapy for DM and its related complications.

OS is a consequence of the redox system disturbance characterized by the notably increased production of reactive oxygen species (ROS). DM has been found out to be an oxidation state of the increased ROS generation. As the level of ROS is elevated in a hyperglycemia environment, the OS mechanism could be activated to trigger hyperglycemia-induced injury, inflammation, and insulin resistance in host organs. OS actively causes cellular injury and death through the physiological function of ROS as second messengers in cell signaling by inducing oxidative damage in cellular constituents, including DNA deterioration and the peroxidation of proteins and lipids. Additionally, OS might directly impair the insulin-producing pancreatic β cell and decrease insulin sensitivity, subsequently resulting in the onset of DM.

The endogenous antioxidant defense systems are frequently constructed to decrease the deleterious ROS in the host body. The antioxidant enzymes including superoxide dismutase (SOD), glutathione peroxidase (GPX), and catalase (CAT) can catalyze the degradation of ROS, while the nonenzymatic antioxidants including glutathione (GSH), polyamine, and bilirubin can directly trap and scavenge free radicals, both resulting in the elimination and reduction of cellular impairments. External antioxidants, such as vitamin C, vitamin E, carotenoids, and various phenylpropanoid derivatives (phenolics, flavonoids, tannins, lignans, and lignins), have been reported with the potential of enhancing the antioxidant defense systems. Gallic acid (GA), as a natural phenic acid from edible plants, has been applied in nutraceutical products as the antioxidant and immunity regulator against infections. The multifaceted health functions of GA could be mainly ascribed to its free radical scavenging ability that helps to prevent or alleviate OS, which is highly involved in DM and diabetic complications. In this review, we focused on the role of OS in the pathological progression of DM and summarize the current evidence about the regulative effect of GA against OS in DM and related complications.

## 2. Oxidative Stress in Diabetes Mellitus and Its Related Complications

### 2.1. Hyperglycemia and Oxidative Stress

It is noted that hyperglycemia could trigger the relevant cell/tissue injury at the sites of diabetic complications via inducing the glycation and glycoxidation of cellular proteins, with the potential to impair organ metabolism. OS pathways can represent a vital connection between glucoregulatory system disruption and tissue injury. Hyperglycemia has been thought of as the primary driving source of chronic or sustained OS in diabetes-related tissue injury. Hyperglycemia conditions can lead to a serious imbalance between the generation of ROS and the antioxidant defense in organs. The hyperglycemia-related source of ROS contains the autoxidation of lipid or glucose and the reduced activity of antioxidants enzymes including SOD, GPX, and CAT, all of which are involved in the antioxidant defense of tissues in diabetic conditions.

The vast array of molecular mechanisms accounting for hyperglycemia-induced excessive ROS production have been well documented, and some important metabolic pathways are regarded as major contributors to the homeostasis of cellular redox, which is potentially associated with further activation of redox-sensitive genes [1], the following of which are emphasized: (1) Depletion of NADPH via the elevated glucose-induced polyol pathway results in less GSH generation from glutathione disulfide, subsequently contributing to ROS production and OS; (2) hyperglycemia-mediated protein kinase C (PKC) elevates the content of diacylglycerol and increased NADPH oxidases (NOX) activity; (3) the upregulated interaction between advanced glycation end products (AGEs) and RAGEs promotes ROS formation through NADPH oxidase and mitochondrial field [2]; (4) the hexosamine biosynthetic activation promotes the increased generation of uridine diphosphate (UDP)-N-acetylglucosamine (GlcNAc) that could induce the elevation of protein glycosylation in the pathological progression of diabetic complications.

### 2.2. Insulin Resistance and Oxidative Stress

Insulin resistance is a primary cause of metabolic syndrome involved in the progression of DM. A high level of ROS found in hepatocytes of obese mice can stimulate insulin resistance and depletion of hydrogen peroxide can improve insulin resistance [3]. The pathological progression of insulin resistance involves the induction of multiple stress-responsive avenues, resulting in cells dysfunction, autophagy, apoptosis, or death in pancreatic β cells that are important to glucose/insulin regulation. Once insulin resistance is developed, stress-responsive pathways such as JNK are activated, and regulatory cytokine expression or direct oxidative damage of interconnected proteins are increased, while this OS can also hamper the insulin sensitivity in response to insulin-related signaling pathways. For example, the increased ROS can lead to dephosphorylating of the insulin receptors by inhibiting the PI3K-Akt signaling pathway, which, in turn, results in the reduction in glucose transporter 4 (GLUT4) translocation for glucose absorption. Meanwhile, excess insulin levels shift PI3K activation to promote the phosphorylation of Rac, a GTPase participating in cell movement regulation via changing the actin cytoskeleton, which eventually extends the activity of NADPH oxidase 4 (NOX4), a potent oxidizing enzyme for ROS production. The increased ROS activates the retromer in a casein kinase-2 (CK2)-dependent manner. The release of retromer from the trans-Golgi network facilitates GLUT4 transportation to lysosomes for degradation, eventually, a high glucose level remains in the oxidative environment. The hyperactive mitochondria also contribute to the overproduction of ROS. ROS-induced nuclear factor-κB (NF-κB), JNK, and p38 MAPK activation occur in mitochondria-induced stress responses through modification of interconnecting target proteins such as glutathione S-transferase P (GSTP), thioredoxin, and NLRPS inflammasome. OS-mediated mitochondrial fission is an important contributor to insulin resistance via modification of the insulin receptor and stress proteins pathways. Mitochondrial dysfunction also shows its role in the cell cycle and apoptosis, and restriction of hyperactive mitochondria can prevent insulin resistance in cells. Thus, the modification of stress-responsive pathways could regulate proximal and distal insulin signaling pathways and subsequently influence insulin resistance in DM progression.

### 2.3. Inflammation and Oxidative Stress

**Figure 1:**
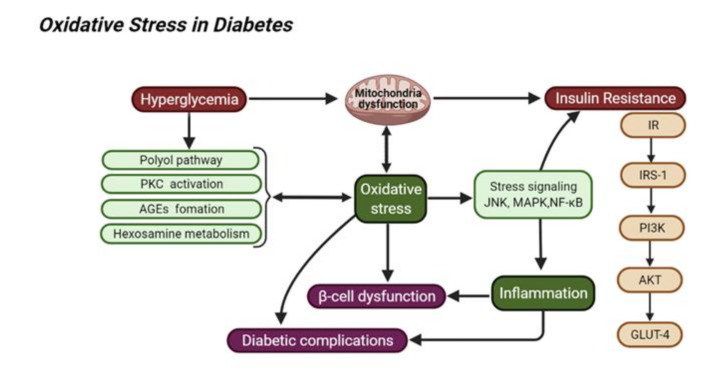
The role of oxidative stress in DM: oxidative stress can promote some important stimuli, such as hyperglycemia, insulin resistance, and chronic inflammation, which may, in turn, aggravate DM.

Chronic systemic inflammation contributes to insulin resistance and the development of DM, which subsequently mediates the aggravation of metabolic complications in DM. The inflammatory response in immune cells such as macrophages can be activated at the site of cell or tissue injury. It is believed that the ROS-induced OS can stimulate a chronic inflammatory response. For instance, excess ROS can accelerate inflammation via activating some transcription factors such as NF-κB and activator protein-1 (AP-1), to mediate the expression of pro-inflammatory cytokines (TNF-α, interleukins-6, and IL-β). In turn, proinflammatory cytokines can provoke the formation of ROS by activating immune cells such as macrophages. The interaction between inflammation and OS might be accounted for the pathological progression of diabetes-associated complications. In part, cellular oxidative damage and inflammatory response triggers apoptosis and/or necrosis during the progression of diabetic complications. Moreover, DM progression can be aggravated through increasing intracellular formation of AGEs and activating the PKC, hexosamine, or polyol pathways. The related metabolic signaling pathways might stimulate the production of ROS, following with the activation of certain inflammatory pathways. Thus, as shown in Figure 1, targeting OR-inflammation pathways might be an attractive intervention for the management of DM and diabetic complications [4].

## 3. Gallic Acid as a Natural Antioxidant

### 3.1. Gallic Acid in Edible Plants

Gallic acid (GA), a natural phenolic acid with a chemical formula C_6_H_2_(OH)_3_COOH, is a common secondary metabolite abundantly distributed in various edible plants including gall nut, tea leaf, oak bark, blueberry, grape seed, rose flower, sumac, witch hazel, the fruit of *Syzygium cumini*, etc. [5]. The biological activities of GA and its derivatives might contribute to the nutritional value of these GA-rich edible plants. For example, extensive studies indicated that tea owned the cholesterol-lowering and antioxidant actions [6]. The health benefits of tea mostly come from the phenolic compounds that illustrated a wide range of pharmacological activities regarding antioxidant, anti-inflammation, and protective properties against severe metabolic disorders, including cardiovascular and neurodegenerative diseases, cancer, hyperlipidemia, obesity, and diabetes. Tea polyphenols containing catechins and GA are known to be enriched in white and green tea [7]. Total polyphenols range from 13.7 to 24.7 g/100 g in green tea and from 10.60 to 25.95 g/100 g in white tea [8]. Epigallocatechin gallate (EGCG), derived from epigallocatechin, and GA are the main bioactive catechins in green and white tea. Accumulating human evidence has shown that the protective properties of tea against serious chronic diseases can be traced back to the potent beneficial effects of EGCG as an antioxidant, anti-inflammation, and mitochondrial function modulator [9]. Additionally, GA and protocatechuic acids as the antihyperglycemic principles were found in *Hibiscus sabdariffa* that is used as a refreshment food drink. Consumption of this drink also has shown beneficial effects on the improvement of hypertension, dyslipidemia, and liver disorders. The antioxidant and antiglycation properties of some edible plants such as *Anacardium humile* St. Hill. has been proven to be associated with the bioactivities of quercetin, catechin, and GA, which could inhibit the glycolytic enzyme activity in RAW264.7 macrophages.

### 3.2. Gallic Acid in Traditional Chinese Medicine

GA has been identified as a bioactive phytochemical component of some medicinal plants, including *Sanguisorba officinalis* L. (Sanguisorbae radix), *Punica granatum* L. (Pomegranate rind), *Terminalia chebula* Retz. (Chebulae Fructus), etc. The bioactive activities of GA and its derivates found in traditional Chinese medicine are associated with the improvement of the metabolic profile in the host following treatment with the related herbal remedy. We provide a summary of GA-rich traditional Chinese medicine in the following sections.

#### 3.2.1. Sanguisorbae Radix (DiYu)

Sanguisorbae radix *(Sanguisorba officinalis* L.), named DiYu in China, is a well-established herbal medicine in Asia for treating diarrhea, duodenal ulcers, enteritis, skin burns diseases, and internal hemorrhage. Additionally, DiYu therapy has been applied for allergic skin diseases, including urticaria, eczema, and allergic dermatitis. DiYu has exhibited anticancer, anticontact dermatitis, antiwrinkle, and bactericidal bioactivities. The mechanism research showed that water extract of DiYu could suppress degranulation of mast bone marrow-derived mast cells induced by IgE/Ag and decrease the TNF-α/IFN-γ-induced inflammation by inhibiting the expression of the phosphorylated JNK and p38 in human keratinocyte cells [10]. Additionally, DiYu has shown antibacterial activity against *Propionibacterium acnes* and antioxidant effects in vitro. GA isolated from DiYu showed the neuroprotection against amyloid-induced neuronal apoptosis [11]. GA, EA, and quercetin from DiYu could inhibit LPS-induced inflammatory activities in macrophage cells. Especially, EA showed the most inhibitory activities and could be attributed as the most potent bioactive constituent in DiYu for treating inflammatory disease [12]. GA, EA and catechinic acid (CA) have been confirmed as the potential chief components accounted for the synergistic therapy of DiYu with 5-fluorouracil on colorectal cancer [13].

#### 3.2.2. Chebulae Fructus (KeZi)

*Terminalia chebula* Retz. is an important herbal plant mentioned in Tibetan and Ayurveda medicine. The widespread clinical pharmacological application in traditional medicine involves treating memory impairment and as an antiaging agent [14]. Meanwhile, the dried fruit of *Terminalia chebula* Retz., named Chebulae Fructus (KeZi in Chinese), is an important herbal remedy for hepatic and intestinal detoxification [15] in traditional Chinese medicine. KeZi can be applied for arthritic disorders due to its radical scavenging activities and anti-inflammatory effects [16]. It has been reported to contain tannin-rich substrates and the highest yield of tannase, and GA has been identified from tannin-rich substrates by fermentation with Rhizopus oryzae and Aspergillus foetidus [17]. Meanwhile, the major components for the antidiarrhea activities of KeZi were identified as GA, EA, 3, 4, 6-tri-O-galloyl-β-d-Glc, and corilagin [18]. GA and chebulagic acid (CA) as KeZi’s active principles could block the T lymphocyte-mediated cytotoxicity. Chebulae Fructus Immaturus (XiQingGuo in Chinese), originated from the immature fruit of *Terminalia chebula* Retz., is also a traditional Tibetan medicine that has shown effective antioxidant action. EA, ethyl gallate, and tri-n-butyl chebulate have been isolated from Chebulae Fructus Immaturus and total tannins of Fructus Chebulae Immaturus and ethyl gallate extracts had shown beneficial antibacterial effects against *Klebsiella pneumoniae* and *Staphylococcus aureus*.

#### 3.2.3. Pomegranate Rind (ShiLiuPi)

Pomegranate Rind (ShiLiuPi in Chinese), originated from the fruit of the tree *Punica granatum* L., is particularly abundant in the hydrolyzable tannins, such as punicalagin that is a chemical large molecule composed of GA and ellagic acid along with a glucose moiety. ShiLiuPi is commonly applied for the treatment of fungal and bacterial pathogens in Uyghur and traditional Chinese medicine. These tannins have been identified as being the chief sources of bioactivity responsible for the significant medicinal properties of ShiLiuPi, including the anti-inflammatory action against peptic ulcer, antimicrobiota response, and the dermal wound healing efficacies. The compounds GA, EA, and punicalagin A&B extracted from ShiLiuPi, could potentially inhibit LPS-mediated NO, PGE-2, and IL-6 release from RAW264.7 cells. GA has shown a broad range of antifungal effects against Candida strains, such as *Candida albicans* and *Trichophyton rubrum*. The mechanism of its antibacterial effects was correlated with the reduction of squalene epoxidase and sterol 14α-demethylase P450 activation in the *T. rubrum* membrane [19].

Additionally, some other GA-rich herbal or formula medicines play important roles in traditional medicinal applications. For example, Chios Gum Mastic is the resin secreted by the trunk of *Pistachia lentiscus* var. chia in the eastern Mediterranean. The oleanonic acid, oleanolic acid, and GA as PPARs modulators involved in the pharmacological effects of Chios Gum Mastic on dyslipidemia, diabetes mellitus, atheromatosis, and neoplasias. Additionally, their synergistic action on PPARs could be one of the main molecular mechanisms through which Chios Gum Mastic exerted its multiple effects [20]. The Ayurvedic traditional preparation Amalaki rasayana is commonly used for treating cardiovascular diseases, inflammatory conditions, and cancers. The known activities of GA, EA, and arachidonic acid compounds found in Amalaki rasayana have been associated with the metabolic profile alteration following supplementation of Amalaki rasayana [21]. Triphala is composed of three dried fruits originating from *Terminalia bellerica* Roxb, *Terminalia chebula* Retz., and *Phyllanthus emblica* Linn. This Ayurvedic medicine has shown its effective treatment on dyspepsia, headaches, and leucorrhoea and its modern pharmacological properties on hypocholesterolemic, anticancer, radioprotective, hepatoprotective, and antioxidant effects. Triphala’s principle constitutes are the tannins and GA found in these three herb plants constituting Triphala. The content of GA present in the three plants was considered as the standardization of Triphala [22].

### 3.3. Gallic Acid as a Natural Bioactive Metabolite

GA is often obtained by the hydrolysis of tannins (gallotannins and ellagitannins) or polyphenol tannic acid [23]. Additionally, GA also could be synthesized by acidic, alkaline, biological, or enzymatic processes. GA could be synthesized by the hydrolysis of tannic acid with an inducible hydrolase of *Enterobacter* spp. [24]. Tannic acid had a high yield in many edible plants, including fruits, berries, and nuts (persimmons, apples, grape, almonds). Ellagic acid (EA), a condensed dimer of GA, can be found in cranberries, blueberries, raspberries, and walnuts, either in the bound formation (ellagitannins) or in the combination with hexahydroxydiphenic acid. EA exhibits a broad spectrum of pharmacological properties including antioxidant, antihyperglycemic effects, as well as regulating apoptosis-inducing activities that are facilitated for treating several human chronic diseases [25].

In humans, hydrolysis of tannins occurs in the digestive tract before absorption or microbial catabolism. Nonenzymatic hydrolysis of gallotannins could release free GA in the colon, and bacterial decarboxylation of gallotannin could produce pyrogallol together with GA [26]. A human pilot trial showed that hydrolysis of gallotannins from Keitt mango could release GA in the gastrointestinal tract and a study of GA production from the hydrolyzable ellagitannins presents in the stomach or small intestine [27], suggesting that tannins might act as a pool of GA derivatives that can be metabolized or facilitated by gut bacterial actions [28].

It has been pointed out that gut bacteria as an important regulator of the healthcare of polyphenols [29] can stimulate tea polyphenol metabolism [30], gut bacteria including *Enterobacter aerogenes* and *Bifidobacterium longum* subsp. infantis have been found to induce EGCG to release EGC and GA [31]. The fermentation of black tea could produce more GA in tea as a result of the 3-galloyl substituted catechins de-esterification either by oxidative degradation or native esterase [32]. GA is nontoxic, with an LD_50_ dose of 5 g/kg in rats, but it is quite evident that rapid metabolism leads to its low bioavailability and poor absorption [33]. GA at the dose of 10 and 20 mg/kg can improve the protective effects of gallic acid in diabetic rats; after oral administration of GA at a dose of 0.3 mmol, about 70% of GA is absorbed and mainly converted into 4-O-methyl-gallate and pyrogallol, and GA was rapidly eliminated with mean maximum concentrations of 2.09 ± 0.22 μmol/L in plasma. A high level of GA and its major metabolites in the urine can be determined within a short time of GA administration, suggesting the excretion of GA is fast in the host metabolism [34].

### 3.4. Structure Modification of GA

To improve GA’s nutritional value, structure modification of GA has been attempted to change the polar nature of this nonbiodegradable and poor-water soluble phenolic compound and promote its absorption into the enterocyte membrane [35]. Nanoformulations could increase their biodegradability and bioavailability [36]. A complexation with hydrogenated soy phosphatidylcholine (HSPC) improves lipophilicity and enhances the bioavailability of GA. The GA–HSPC complex nanoformulation increased the antioxidant capacity against CCL_4_-induced hepatotoxicity [37]. The soybean lecithin–GA complex exerted hepatoprotective effects on alcohol-induced hepatic damage [38]. GA could be utilized for the synthesis of gold nanoparticles in drug delivery for breast cancer treatment [39]. GA–hydroxypropyl-β-cyclodextrin complex showed higher antifungal activity than GA and imparted better solubility for treatment of *Candida albicans* biofilm [40]. The enhanced antioxidant properties of GA-grafted chitosan have been confirmed in the real bulk oils [41]. Conjugation of GA with polyamidoamine dendrimer or phosphatidylcholine increased its stability and controlled sustained release of GA into the host. A GA–chitosan nanoformulation considerably improved the thermal stability as high as 140 ℃, with its antioxidant functions remaining intact. The synthesized gemini gallate interfacial antioxidant showed superior antioxidant activity and extraordinary radical scavenging performance in the O/W emulsion, compared with monogallate [42].

## 4. Mechanism of Gallic Acid’s Antioxidant and Anti-Inflammation

**Figure 2:**
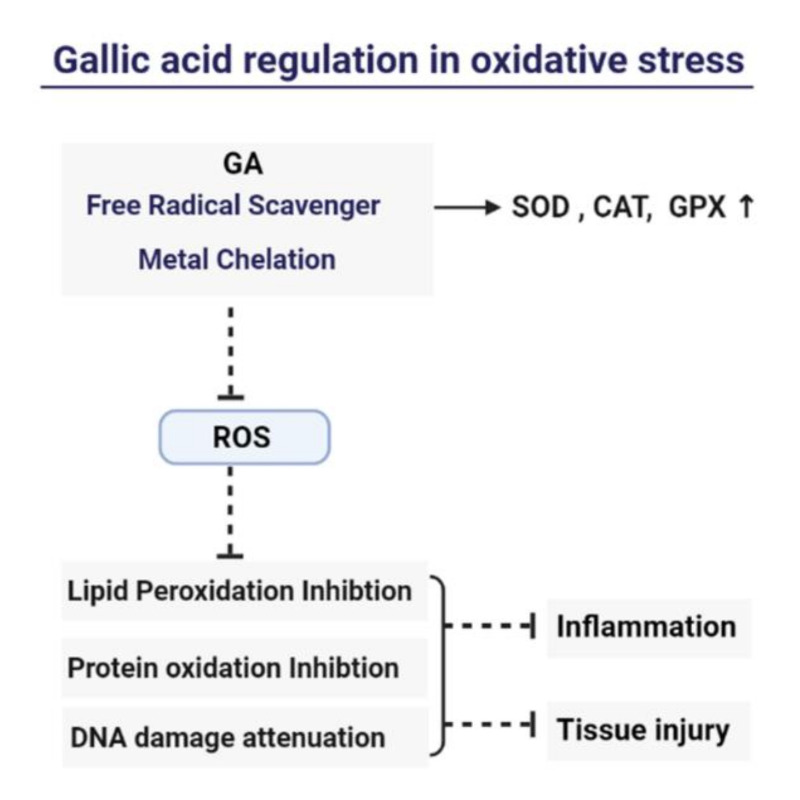
Regulation of oxidative stress by gallic acid: GA can exhibit the free radical scavenging ability and show the beneficial effects on the cell redox pathways such as GPX/GSH system. These actions suppress the generation of ROS, which, in turn, reduce lipid peroxidation, protein oxidation, and DNA damage, finally inhibiting the inflammatory response and tissue injury.

The phenolic hydroxyl groups are able to interact with the benzene ring of molecules endowed with the ability to generate free radicals, and thus, they are allowed to mediate radical oxidation. Additionally, phenolic chemicals could chelate transition and subtract metal ions that promote free radical damage. Phenolic structures with hydrophobic phenyl rings and phenolic hydroxyl groups are facilitated to form hydrogen-bonding interactions, which could also collaborate with proteins such as cytochrome P450 isoforms, cyclooxygenases, lipoxygenases, and xanthine oxidases. Thus, they could inhibit some oxidative enzymes involved in radical production (i.e., advanced oxidation protein products (AOPPs), malondialdehyde (MDA), oxidized low-density lipoproteins (oxLDLs)). Except for its scavenging ability, GA also may exhibit pro-oxidative activities as shown in Figure 2. Indeed, the biological activities of GA might rely on its behaviors as either an antioxidant or a prooxidant. GA showed potent antioxidant activities through the formation of stable semiquinone FRs that lead to the attenuation of FRs’ deaminating ability. Many studies proved that its potent antioxidant activities of scavenging free radicals are involved with Ferric-reducing antioxidant power (FRAP) and 2,2-diphenyl-1-picrylhydrazyl (DPPH) or oxygen radical absorbance capacity (ORAC) [43]. Due to GA having the phenolic hydroxyl group, the good hydrogen-donor can react with ROS or reactive nitrogen species (RNS) to block over-production of damaged FRs, including peroxyl radicals, hydroxyl, and superoxide radicals, and peroxynitrite radicals by avoiding the nitration of tyrosine. Meanwhile, mechanism related to regulating OS is associated with the biosynthesis of GPX and GSH. GA inhibited aflatoxin biosynthesis in *Aspergillus flavus* via the regulation of CreA and FarB, which is involved in the attenuation of OS via the GSH- and thioredoxin-dependent systems [44]. Additionally, delphinidin and its metabolite, GA, could increase intracellular GSH in endothelial cells [45]. GA could prevent the tissue oxidative damage exposed to dibutyl phthalate or acute ketamine. GA revealed radioprotective properties against cell damage caused by the adverse side effects of cancer radiotherapy [46]. Supplementation of GA (100 mg/kg) reduced γ-radiation-induced cellular DNA damage in blood leukocytes, bone marrow cells, and splenocytes of whole-body irradiated mice. The radiation-induced decrease in GPX and GSH levels was restored by the supplementation of GA in various tissue of irradiated mice, with the inhibition of the peroxidation of membrane lipids, consequently leading to lower weight loss and mortality after γ irradiation [47].

GA could inhibit the growth of HCT-15 colon cancer cells via regulation of ROS-dependent cellular apoptosis, and it is known that GA-induced A549 lung cancer cells death is related to ROS increase and GSH depletion. Additionally, GA could modulate the gene expression of proapoptotic (Bax and Bad) and antiapoptotic (B-cell lymphoma-extra large (Bcl-xL) and Bcl-2) proteins. The antitumor action of GA was mediated by the reduction of Bcl-2 expression that reversely promotes the Bax translocation into mitochondria to produce cytochrome-c and subsequent cellular apoptosis [48,49]. Meanwhile, GA can exert its potent inhibition on cancer cell invasion and metastasis by downregulation of algogenic substances such as matrix metalloproteinase -2/9 (MMP-2/9) via the anti-inflammatory response [50]. GA could inhibit EGFR/Src-mediated Akt and ERK activation, resulting in decreased expression of p65/c-Jun-mediated DNA looping, thus reducing the expression of MMP-9 protein in EGF-treated MCF-7 cells [51]. GA’s inhibition on MMP-2/9 also involved the suppression of the NF-κB signaling pathway in gastric adenocarcinoma cell metastasis and the cytoskeletal reorganization [52]. GA was also confirmed to restore the antioxidant and inflammatory status to the normal levels via nuclear factor erythroid 2-related factor 2 (Nrf2) activation [53,54]. GA exerted its inhibitory effect on proptosis and NLRP3–NEK7 interaction dependent on the Nrf2 signaling [55]. GA could also modulate the ERK/Nrf2-induced antioxidative signaling pathway, and the potential mechanism showed that GA possibly competed with Nrf2 for binding to Keap1 [56]. The effects of GA reversed the methylglyoxal-induced changes in albuminuria, MDA, Nrf2, miR-192, and miR-204 upregulation, as well as decreased the levels of SOD, CAT, and glyoxalase1 expression in methylglyoxal-induced diabetic nephropathy.

The other mechanism by which GA shows its potent anti-inflammation is the scavenging of NO and the inhibition of NO synthase and inducible nitric oxide synthase (iNOS) activities, which could impede the induction of TNF-α, lipopolysaccharides (LPS), IL-6, and interferon-α (IFN-α) expression. GA improved the oxidative and inflammatory status that promoted the recovery of the neuronal morphology in the hippocampus [57]. It exerts anti-inflammatory effects against metabolic disorders such as insulin resistance, dyslipidemia, and obesity [58] by modulation of adipocyte–macrophage crosstalk [59]. GA-treated obese mice had decreased expression of IL-6, iNOS, cyclooxygenase-2, F4/80, and sterol regulatory element-binding transcription factor-1 (SREBP-1) in adipose tissue. GA could suppress the activation of the p65-NF-kB and IL-6/STAT3 pathways in adipose and decrease adipogenesis by inhibiting the expression of monocyte chemoattractant protein-1 (MCP-1) and increasing that of adiponectin and peroxisome proliferator-activated receptor-γ (PPAR-γ). GA also reduced inflammatory response mediated by the co-culture of adipocytes with macrophages. Thus, GA suppresses adipocyte hypertrophy and inflammation caused by the interaction between adipocytes and macrophages, thereby improving metabolic disorders such as insulin resistance and dyslipidemia [58].

## 5. Gallic Acid for Diabetic Therapy

GA and its derivates function as potent antioxidants and free radical scavengers, with the potential to modulate inflammation, apoptosis, or oxidative stress in different pathophysiological situations. GA has been reported to possess antihyperglycemic potential through its antioxidant and anti-inflammatory properties [60]. GA from *E. officinalis* fruit juice facilitated insulin sensitivity and glucose homeostasis in adipocytes [61]. Mechanistically, PPAR-γ and C/EBPs activation simultaneously promoted GLUT4 translocation in adipocytes. Moreover, GA could increase insulin sensitivity by regulation of Akt and AMPK signaling pathways, thus showing evidence of dual activation of Akt and AMPK by fruit juice of *E. officinalis* [61]. The findings indicated that PPARγ, Akt, and AMPK activation contributed to the antidiabetic action of GA [62]. Additionally, the antidiabetic effects of GA could be mediated via regulation of TNF-α and adipocytokines expression. GA improved the function of the β cells by inhibiting caspase-9-related cellular apoptosis. AGE/ALE end products were accountable for the development of different oxidative-based diabetic complications such as nephropathy. GA could reverse glyoxal-induced renal cell viability reduction, membrane lysis, ROS formation, lipid peroxidation, mitochondrial membrane potential collapse, and lysosomal membrane leakage, which lead to advanced glycation inhibition. Diabetic patients often suffer from chronic, impaired wound healing, which facilitates bacterial infections and necessitates amputation. GA could accelerate cell migration of human keratinocytes and fibroblasts and activate factors known to be hallmarks of wound healing, such as focal adhesion kinases (FAK), c-Jun N-terminal kinases (JNK), and extracellular signal-regulated kinases (Erk), underpinning the beneficial intervention of GA in wound repair resulting from diabetes. This polyphenol compound modulated different antidiabetic signaling pathways through its antioxidative potential in diabetic complications, and we summarized some potential antidiabetic herbal and edible plants that mentioned GA as a major contributor, as shown in Table 1. Meanwhile, GA could improve cardiac complications, diabetic nephropathy (DN), and neuropathy, in addition to preventing OS-induced liver and renal injury in the diabetic state.

### 5.1. Diabetic Cardiovascular Diseases

GA plays a significant role in decreasing critical diabetic complications such as diabetic cardiomyopathy. GA improved cardiac arrhythmias and cardiac electrophysiology during reperfusion in the alloxan-induced diabetes. It improved hypertrophy and left ventricular dysfunction of ischemia–reperfusion damage in alloxan-induced diabetes mellitus. GA could also improve endothelial dysfunction and hypotension in diabetes via upregulation of plasma miR-24 and miR-126 levels.

### 5.2. Neurodegeneration

GA is a well-known antioxidant compound that has shown neuroprotective effects on neurodegeneration and neurotoxicity. The GA-induced FRs damaging interference has been attributed to its protective action against neurodegeneration diseases. It is reported that GA antagonized semen amyloid fibrils and reduced HIV-1 infection [83], and it made interaction with α-synuclein to block amyloid fibrils formation in the progression of Parkinson’s disease [84]. GA has shown its antioxidative stress against hippocampal neurodegeneration in type 2 diabetic rats. Meanwhile, the hippocampus of GA-treated diabetic rats showed an improvement of the Bax/Bcl-2 ratio, as well as a decreased inflammatory response. GA reversed the increased lipid peroxidation and decreased GSH levels in the hippocampus and prefrontal cortex of streptozotocin-treated diabetic animals.

### 5.3. Diabetic Nephropathy

GA may inhibit SA-induced kidney and liver toxicity through scavenging reactive free radicals and increasing intracellular antioxidant capacity.GA improved methylglyoxal-induced DN via amelioration of OS and microRNAs related to endoplasmic reticulum stress and renal fibrosis. GA treatment significantly reduced the levels of serum creatinine, blood urea nitrogen, and albumin, as well as improved creatinine clearance and decreased the expression of TGF-β1 in type I diabetic nephropathy rats.

### 5.4. Liver Injury

GA ameliorated Aflatoxin B1-mediated hepatorenal dysfunction by suppressing OS, inflammation, and enhanced apoptosis [85]. GA mitigated diclofenac-induced liver toxicity by modulating antioxidant defense system and suppressing IL-1β gene expression in male rats [86]. Oral GA reduced hepatic lipogenesis and improved hepatic steatosis and metabolism in obese mice [87]. Gallic and ferulic acids exhibited hepatoprotective and antioxidant effects against thioacetamide-induced liver fibrosis in rats. These effects are mediated through enhancement of antioxidant activity, inhibition of TGF-β1/Smad3 pathway, and differential regulation of the hepatic expression level of miR-21, miR-30, and miR-200 [88].

### 5.5. Clinical Application

Increasing evidence has been provided to support the findings that the intervention of natural phenolic antioxidants have shown potent beneficial effects, which, at present, have been clinically adopted for DM management, such as oil palm phenolics for uncontrolled insulin-treated type 2 diabetes mellitus (NCT02532101), flavonoids for essential hypertension and type 2 diabetes (NCT03722199), cacao and carob for type 2 diabetes (NCT04383639), spiced meat patty for inflammation in men with type 2 diabetes (NCT01076829), extra virgin olive oil (NCT04025281), cinnamon and cassia bark (NCT00479973), bilberry (NCT01245270), stingless bee honey for diabetic wound bed preparation (NCT04849143), and yerba mate extract for cardiovascular diseases in diabetic patients (NCT02789722). GA and its related edible plants have been specifically credited for reducing systemic inflammation and OS-associated metabolic complications including DM and related complications in humans. Jaboticaba consumption could improve serum antioxidant capacity and significantly decrease serum insulin in humans. When the subjects with metabolic syndrome consumed the test meal with beef burgers prepared with wine grape pomace flour, their fastened glucose and insulin resistance were improved, as with the plasma antioxidant levels. Green tea polyphenols as an effective antioxidant could lower OS, thereby ameliorating skin texture and integrity. Moreover, the safety evaluation of a skin test showed that GA used in 31 allergic contact dermatitis patients reactive to P paraphenylenediamine and/or paratoluenediamine hair dyes is safe.

## 6. Conclusions

The pathophysiological progression of diabetes and associated complications have been found to be associated with different complex mechanisms, and hyperglycemia, insulin resistance, inflammation, and OS are commonly considered as the major causal factors implicated in the worsening of diabetes-associated perturbations. Thus, in addition to controlling the blood glucose and insulin levels, amelioration of inflammation and OS may be another basic interference made to improve cellular function in the diabetic state. At present, only a few effective antioxidant therapies are available to improve the metabolic syndrome of diabetic patients. To date, polyphenol-rich-food consumption has been associated with several multitarget antioxidative activities. Particularly, GA, the common chemical entity of polyphenols, has shown promising results in treating DM and its related complications. Some natural products including GA have been shown to ameliorate high glucose levels, insulin, inflammation, and OS in diabetic complications through activation of multiple effective pathways such as radical production, GPX/GSH system, NO/iNOS generation, AGE/ALE, JNK/ERK, GLUT4, and Nrf2 pathways. Certainly, the preclinical data summarized in this review supported the health benefits of GA or its derivatives in preventing diabetic-associated complications. Other interesting questions raised in this review highlight the fact that the in vitro multitarget properties of GA often do not coincide with in vivo results, probably due to poor oral bioavailability, thus indicating the demand to investigate its structure modification with the aim to improve its absorption and the distribution in mammals, which thereby enhances its healthy properties and efficacy when applying new formulation. Convincing evidence is now available from previous studies that prove the role of oxidative stress in the onset and the course of diabetes and complications, as well as the beneficial effects of GA in these diseases. Future research is still required for exploring the antioxidative effects of GA against diabetes-associated complications in human subjects to confirm its therapeutic potential. This can be further complemented with experiments exploring its combination use with current glucose-lowering therapies to investigate whether it possesses therapeutic benefit as an adjunct therapy. Thus, the presence of an abundant amount of GA in edible and herbal plants provide the evidence for the antidiabetic potential of the related plants and delineate the antioxidative and anti-inflammation in GA-mediated antidiabetic activity, thus providing potential therapy for diabetes [61].

## Figures and Tables

**Table 1:** Potential natural antidiabetic herbal and edible plants containing GA.

The Natural Plants	The Identified Phenolic Derivatives	Pharmacological Activities
*Anacardium humile* A.St-Hill. [63]	quercetin, catechin and GA	Antioxidant, antiglycation/α-mylase inhibitors
*Bergenia stracheyi* (Hook. f. & Thoms.) Engl. [64]	GA, 11-O-galloylbergenin, (-)-epicatechin 3-O-gallate	Antidiabetes, antioxidant
Tea leaves including black, green, and white tea [7]	caffeic acid (CA), GA	Colesterol-lowering, antioxidant
Chios Gum Mastic	oleanonic acid, oleanolic acid, GA	PPARs modulators,
*Cochlospermum regium* (Schrank) Pilg. Root [65]	GA, CA and ellagic acid	Antioxidant, antidiabetes, antiglycation, anticholinesterase
*Citrus reticulata* Blanco [66]	total phenolic chemicals	α-amylase and α-glucosidase inhibitory
*Delonix regia* (Bojer ex Hook.) Raf. [67]	quercetin, GA, CA, cinnamic acid, ferulic acid, and p-coumaric acid	Antioxidant, hypoglycemic, and hypolipidemic activities
*Delonix regia* (Bojer ex Hook.) Raf.	quercetin, gallic acid, caffeic acid, cinnamic acid, ferulic acid, and p-coumaric acid	Hypoglycemic, antioxidant, and hypolipidemic activities.
*Entada spiralis* Ridl. Stem Bark [68]	GA, (+)-catechin, (-)-epicatechin.	Antioxidant activity
*Eugenia punicifolia* (Kunth) DC.	myricetin-3-O-rhamnoside, quercetin-3-O-galactoside, quercetin-3-O-xyloside, quercetin-3-O-rhamnoside, kaempferol-3-O-rhamnoside, phytol, gallic acid, and trans-caryophyllene	Antidiabetic activity
*Eugenia uniflora* O. Berg	ellagic acid, GA and rutin	Antioxidant and anti-inflammatory activities
*fermented legumes*	gallic acid, catechin, caffeic acid, epicatechin, rutin, isoquercitrin, quercitrin, quercetin and kaempferol	Antidiabetic and anti-acetylcholinesterase activities
*Gymnema montanum* (Roxb.) Hook.f.	gallic acid, resveratrol, and quercetin	Protection against lipid peroxidation
*Hibiscus sabdariffa* L. [69]	GA and protocatechuic acids	Improvement of diabetes, hypertension, dyslipidemia,
*Linum usitatissimum* L. Seeds [70]	GA	Antidiabetic activity
Mango (*Mangifera indica* L.) peel [71]	ferulic acid, protocatechuic, chlorogenic, gallic, vanillic, and caffeic acids	Antioxidant, anti-inflammatory, antidiabetic activities, inhibition of α-amylase and α-glucosidase
*Momordica cymbalaria* Fenzl ex Naudin [72]	gallic acid and rutin	Antidiabetic and improvement of insulin resistance
Mulberry leaves [73]	GA	Modulation of insulin and inflammatory signaling
*Senecio biafrae* (Oliv. & Hiern) C.Jeffrey leaves [74]	GA, chlorogenic, caffeic acid, rutin, quercetin, and kaempferol.	Antidiabetic activity
*Syzygium cumini* (L.) Skeels kernels powder and fruits [75]	Myricetin, catechin, quinic acid, chlorogenic acid, ellagic acid, catechin, gallic acid, and caffeic acid	Antioxidant, anti-inflammatory, anticancer, antidiabetic, antibacterial, antifungal activities
Triphala Rasayana	GA, ellagic acid, chebulic acid, chebulinic acid, methyl gallate	Antidiabetes, anticonstipation, antiobesity
Jikan Mingmu Drop [76]	GA, ellagic acid,	Reduction of dry eye syndrome
*Tamarix stricta* Boiss [77]	GA	Antidiabetic activity via autophagy
*Terminalia paniculata* Roth Bark [78]	GA	Antidiabetic activity
*Punica granatum* L. [79,80]	punicalagin and ellagic, gallic, oleanolic, ursolic, and uallic acids	Antidiabetes, antipain in diabetic neuropathy
Pinus gerardiana Wall. ex D.Don [81]	GA	Inhibition of α-amylase, antioxidant, antihyperlipidemic antidiabetes
*Ziziphus mauritiana* Lam. leaves [82]	rich in polyphenols	Antioxidative stress in diabetes
